# Keeping silent or running away. The voices of Vietnamese women survivors of Intimate Partner Violence

**DOI:** 10.1080/16549716.2020.1863128

**Published:** 2020-12-30

**Authors:** Raquel Herrero-Arias, Anh Ngoc Truong, Gaby Ortiz-Barreda, Erica Briones-Vozmediano

**Affiliations:** a Department of Health Promotion and Development, Faculty of Psychology, University of Bergen, Bergen, Norway; b Research Group of Child Welfare, Equality and Social Inclusion, University of Bergen, Bergen, Norway; c Gender Equality Department, Ministry of Labour, Invalids and Social Affairs of Vietnam, Hanoi, Vietnam; d Nursing and Physiotherapy Department and Faculty, University of Lleida, Lleida, Spain; e Healthcare Research Group (GRECS), Biomedical Research Institute (IRB), Lleida, Spain

**Keywords:** Intimate partner violence, abused women, qualitative study, Vietnam, help seeking

## Abstract

**Background**: Legislative initiatives have been implemented to fight against Intimate Partner Violence (IPV) and offer protection to its survivors in Vietnam. However, this type of violence is relatively common in the country, where broader structural inequalities, like poverty and the system of male dominance, increase women’s vulnerability to IPV.

**Objective**: This study aimed to explore the strategies that Vietnamese IPV survivors take to cope with the abuse from their partners and maximize their safety and wellbeing.

**Methods**: Qualitative in-depth interviews were conducted with eight women survivors of IPV who lived in one of the Peace House Shelter in Hanoi. Participants were recruited through the shelter. Data were collected using semi-structured interviews and analyzed using qualitative content framed by the theoretical concept of the patriarchal bargain.

**Results**: The IPV survivors in our study took two main strategies to cope with IPV: keeping silent and/or leaving the abuser. Leaving was a challenging strategy because it required support from others, something that was difficult to find due to the social stigma associated with divorce and the normalization of violence in intimate relationships. This was specially the case for participants coming from rural areas who did not count on a social network in the city where the shelter is located. The women strategized within a complex set of structural constrains like poverty, cultures of honor, social stigma, and traditional gender roles. As active agents, they decided whether challenging the patriarchal system would optimize their life options. Motherhood also played a crucial role in women’s decisions regarding IPV.

**Conclusion**: A strategy of conformity like silence can be a tactic for women to cope with a system of male dominance while navigating complex structural inequalities. To better address IPV in Vietnam, interventions should be sensitive to the structural gender inequalities within family and societal contexts.

## Background

The World Health Organization defines Intimate Partner Violence (IPV) as ‘behavior by an intimate partner or ex-partner that causes physical, sexual or psychological harm, including physical aggression, sexual coercion, psychological abuse and controlling behavior’ [1]. Scholarship has found that unemployment, low educational attainment and income, and weak social support increase women’s vulnerability to IPV [2], which is, generally, more common in low and middle-income countries [3,4]. Because of its high worldwide prevalence and serious consequences for survivors and their children, IPV is recognized as a major public health and human rights problem [5].

The recognition of IPV as a problem in more traditional and patriarchal societies with gender norms like Vietnam is relatively new and has remained normalized and neglected until only recently [6–10]. A report on domestic violence against women by the Vietnam General Statistics Office and United Nations Population Fund (UNFPA) shows that 58% of ever-married Vietnamese women have experienced at least one type of violence (physical, emotional or sexual) from their partners during their lifetime [11]. The corresponding figures for physical, emotional, and sexual violence were 32%, 54%, and 10%, respectively. According to this report, 87% of women suffering from IPV did not seek any help from public services. Among the reasons listed, fear of the shame associated with leaving the abuser and beliefs that normalize violence in romantic relationships stood out. Regarding the few cases of IPV that were reported, 61% were referred to mediation/reconciliation, 12% resulted in criminal charges and only 1% led to convictions. In a context of high rates of IPV and of the mental problems and serious injuries associated with it, there is a need to improve the health care services and the support that is available for women suffering from IPV in Vietnam [8].

Vietnam is an example of a country whose government has become more aware of the problem of IPV and has legislated to safeguard the safety and protection of women suffering from this kind of violence. The 2006 Law on Gender Equality enshrined the principles of gender equality in social and family life and assigned key responsibilities to different government agencies, who were mandated to focus on achieving gender equality. A year later, the government took a further step towards the prevention of IPV and the facilitation of access to social, health and legal services to the survivors with the Law on Domestic Violence Prevention and Control. This law legislates the duties of individuals, families, institutions, and organizations in relation to the prevention and control of IPV and the support of women and children. It includes education measures for repeat offenders, national hotlines for women who are experiencing IPV, and the implementation of quality services for women and children survivors of IPV, like shelters that can be operated both by domestic and international non-governmental organizations (NGOs) and government agencies. Finally, the Marriage and Family Law (2014) includes regulations on gender discrimination, such as the different minimum age of marriage for women and men, and provides for the principle of gender equality in entitlements related to property in divorce and widowhood. In alignment with the Government’s efforts to address IPV and human trafficking, social services and non-public institutions provide shelter for survivors of IPV, sexual assault, and human trafficking through a network of roughly 70 shelters [12].

Despite efforts to fight against IPV, broader structural disadvantages like poverty and the Vietnamese system of male dominance play a decisive role in increasing women’s vulnerability to IPV [13]. The voices of IPV survivors can shed light on the strategies they take to cope with abuse from their partners while confronting structural barriers in Vietnam. Framed by the theory of women’s bargaining under classic patriarchy [14] and drawing on in-depth interviews with women who experienced IPV, this study aims to explore the strategies taken by Vietnamese IPV survivors in the process of coping with abuse from their partners while maximizing their safety and wellbeing.

### Women bargaining under the Vietnamese patriarchy

Drawing on the work of Kandiyoti [14], this article understands that women manage different forms of male domination and maximize their security and life options by adhering to prevailing gender rules that constrain their freedom. Patriarchal bargains are the coping mechanisms that women engage in to optimize their wellbeing and survival within male dominance systems. These do not challenge the patriarchal system but reinforce male dominance and female subordination. Individual women might decide to bargain when they foresee that they will benefit more from adapting to the system rather than from challenging it.

Kandiyoti explored the patriarchal bargain in two systems of male dominance: 1. Sub-Saharan Africa 2. Middle East and South and East Asia. In the latter, which includes the societies of the ‘patriarchal belt’ like Vietnam, classic patriarchy prevails, and women are subordinated to all men and older women. In this male dominance system, which is characterized by the women inheriting authority from more senior women [14], women strategize for their security with conformist actions like becoming oppressors of younger women in extended families or using their unremunerated labor in the household in exchange for their husbands and sons’ affections and the maximization of their security. Women use such coping mechanisms, which do not resist female subordination, because it allows them access to the patriliny through their male offspring. This gives them power over younger women in their role as mothers-in-law. According to traditional Vietnamese culture, married women must live with their in-laws and learn the new rules, routine, and lifestyle from husbands and mothers-in-law [15]. Following Confucian traditions, women are subject to a moral code of obedience to men (fathers, husbands, sons) [16,17]. This tradition promotes an idealized vision of a womanhood based on self-sacrifice, maintenance of family harmony, housework, appearance, conduct, and gendered separation of the private-public spheres [13,18,19]. In this context, motherhood guarantees, to some extent, a higher position for women within the family [16].

The traditional Confucian ideas and communism’s more egalitarian norms have resulted in a ‘mix of patriarchal and egalitarian gender norms and attitudes’ [20]. Today, traditional gender patterns are still common in Vietnamese families where men play a leading role in the family-marriage relationship [21,22]. Women have a key role in unpaid care work, and there is a preference to having sons that persists, and results in a high sex ration at birth [22]. The weight of traditional gender norms, like female sacrifice and endurance, shapes women’s thinking around their sexual and reproductive health. In this context, Vietnamese women seem to recognize their roles and responsibilities, rather than their rights [20].

## Method

### Study setting

The present study was conducted in 2015 in a Peace House Shelter (PHS) for children and women survivors of IPV located in Hanoi. The shelter can host a maximum 40 residents, who are offered kindergarten services, medical care, psychological counselling, legal aid, educational and vocational training, and entertainment activities. A manager, as well as social workers, security guards, and housekeepers make up the staff of this agency.

The PHS model includes two shelters in each of their locations: one for human trafficking survivors, and another one for children and women survivors of IPV. Established in 2007 in a collaboration between the Centre for Women and Development (CWD) and the Spanish Agency for International Development, these shelters provide accommodation for women and children in three locations and are the most popular ones in the country.

### Study design

This study has a qualitative design, and data were collected using in-depth, semi-structured interviews.

### Study participants

Eight residents who had experienced physical and emotional abuse from their husbands agreed to be interviewed for the purpose of this study. At the time of data collection, six of them were living in the PHS with their children and two had already left (residents 1 and 2). The women who had already left received reintegration support from the PHS while they continued to work and raise their children without their husbands. Participants were between 20 and 56 years old and had diverse social and economic backgrounds (Table 1).

**Table 1.** Sociodemographic information of informants (n = 8)

Pseudonym	Age	Occupation	Husband’s occupation	Place of origin	N. Children	Length of IPV
Resident 1	42 years	Waitress	construction worker	Rural area	3	4 years
Resident 2	35 years	Tailor	Unemployed	City	1	8 years
Resident 3	24 years	College student	doctor	Rural area	1	2 years
Resident 4	30 years	No steady job	Not answered	City	2	2 years
Resident 5	56 years	Farmer	Farmer, construction worker, and motorbike taxi driver.	Rural area	3	38 years
Resident 6	28 years	Works in a bank	Soldier	Rural area	1	2 years
Resident 7	40 years	Businesswoman	Not answered	Rural area	2	12 years
Resident 8	21 years	Working in ice-tea shop (in-laws’ business).	Unemployed	Rural area	1	2 years

### Data collection

Before data collection began, ANT introduced the aims of the study to the manager of one of the two PHS located in Hanoi, who granted her access to the shelter for IPV survivors. Taking into consideration the ethical implications of interviewing IPV survivors, ANT received support from PHS in the way of training to conduct the interviews sensitively and support from the social workers, who followed up and supported the participants along the research process. Participants provided informed consent and were ensured confidentiality and that participation was voluntary and could withdraw at any time without consequences. Interviews occurred at a place of their choosing, like the shelter, a cafeteria or a counselling room at the CWD.

Interviews lasted between 90 and 150 minutes and questions were open, addressing women’s experiences with the supporting services, their plans for life after leaving the PHS, their experiences of IPV, their responses to the abuse and accounts of seeking help. The interviews were audio-recorded and transcribed verbatim by ANT.

### Data analysis

Qualitative content analysis as described by Graneheim and Lundman [23] was performed to identify patterns in the women´s accounts. The transcripts were coded line by line following an inductive approach and focusing on its manifest content. First, meaning units that referred to the major content areas were identified. In the next step, meaning units were condensed and coded. Codes were grouped into categories, and themes and sub-themes that connected the underlying content in a group of categories were identified (Supplementary).

## Results

Women used two main strategies when dealing with IPV. The first was ‘keeping silent’ and included the sub-categories of keeping the family together and fear of being ashamed and blamed. The second one was ‘leaving the abuser’. This included seeking help from family and friends, seeking help from PHS, and seeking and taking others’ advice. Tables providing illustrative quotes of these accompany each section (Tables 2–6).

**Table 2.** Theme ‘keeping silent’. Subtheme ‘keeping the family together’

Resident	Selected Quotes
Resident 7	[…] I thought I must strive for my son. I had to accept [violence] in order to keep a full family for him.
Resident 8	If I had divorced my husband, I would not have known where to go … [.]. If I had moved out, my child would also have struggled. […] I also accepted this because I love my daughter. I didn’t know where I could take care of my child, where I could go. […]
Resident 3	If I had divorced my husband, I would have taken care of my child alone. […]I had not had a job yet. I had thought he could buy a house and a car by himself. He also has a stable career, so I believed he was the one I could depend on to raise my child.

**Table 3.** Theme ‘keeping silent’. Subtheme ‘fear of being ashamed and blamed’

Resident	Selected Quotes
Resident 8	I was afraid he’d be in jail again. It’s true that wife-beating breaks the law. Therefore, I was afraid. He had already stayed in prison one time, so it was easy for him to be put back again, and that would bring shame to my family again. […] Also my parents were ashamed of what people said about my marriage. And I felt shamed with everyone. I also did not share anything with my friends.
Resident 7	I think it [keeping silent] is because of worrying for their children and thinking of their families. We [women suffering from IPV] also think of our honor. We have a reputation as married women. I have to say that if we divorce, we would feel shame in front of other people.
Resident 1	I had lived with violence for a long time in the past. I had seen my father beat my mother. So, it affected me […] […] I convinced myself that my husband could beat me. […] When I told my father (about the abuse), he said, ‘He is a husband, so he has the right to beat and scold his wife’.

**Table 4.** Theme ‘leaving the abuser’. Subtheme ‘seeking help from family and friends’

Resident	Selected Quotes
Resident 5	I ran to seek help from my relatives. I asked them to stay and hide in their houses after he vented his anger, and I would return to my home afterwards.
Resident 3	My close friends couldn’t help me very much. I don’t have any close friends in the city (where I lived with my husband). They are poor and live in a rural area.
Resident 7	There was one day that he (husband) went to the hair salon to search for me. So, I ran to hide in my neighbor’s toilet. After he left the salon, I came to my acquaintance’s house and hid in their house for half a day.
Resident 4	He (husband) scolded my parents. He put death curses on my parents. He said he wished they would have a car accident or a train accident. All this was because I asked them for help.
Resident 5	I quickly shut the door, ran upstairs to my sister-in-law’s room, and told her that my husband wanted to stab me. His sister was so afraid of him too.

**Table 5.** Theme ‘leaving the abuser’. Subtheme ‘seeking help from the peace health shelter’

Resident	Selected Quotes
Resident 6	Today, he could scold me, so tomorrow he would beat me, and the day after tomorrow he would kill me. I had to leave him, and I needed some help for that.
Resident 1	My child was not a resident in this city, so it was very difficult to be a pupil in a school in the city due to the school systems, but the PHS supported us so I am satisfied.
Resident 8	I did not think the PHS and counselling service are free, and this was one reason I did not seek help earlier.
Resident 2	Each time my husband took my son out (to motels), he (my son) was always sick and scared. […] He said to me ‘Mommy, there was a tottering giant and it juts out the tongue to tease me’. And he started to point this place … that place. I was really scared. My son is really afraid of his father. So every time, he just remembered that his dad was taking a knife and chasing him and his mother. This made me realize that I needed to seek help from the PHS.

**Table 6.** Theme ‘leaving the abuser’. Subtheme ‘seeking and taking others’ advice’

Resident	Selected Quotes
Resident 1	I told my mother (about the situation), I asked for her advice. She sat and consoled me. She said only one sentence, ‘In modern life, you have the right to decide, and do not be like me who wasted half of my life’.
Resident 2	When I shared everything with her [her husband’s aunt], she advised me that I should try to take care of my child in a good way, that I should divorce my husband. She would never want to advise me to divorce her nephew if there were other ways to escape: ‘You should do something good for your child and you should do what is good for yourself. Let’s do it’.
Resident 4	My child and I looked pitiful. It was midnight, and my hair was ruffed because he had beaten me. I didn’t want to live anymore. I crossed the street and waited for a car accident. A taxi driver saw me holding my baby with one arm, and holding my other child’s hand while walking, and he took my child and me to the street [CWD’s address] after giving me some advice.

### Keeping silent

When informants reflected on the strategy of ‘keeping silent’, they discussed the reasons that motivated them to take this decision.

#### *Keeping the family together* (Table 2)

All participants were mothers and the majority them (6) were still living with their children at the time of data collection. In their accounts, motherhood was an element that played a crucial role in women’s decision-making regarding IPV. Informants highlighted that being raised in a family with both parents would be optimal for their children’s development, which they identified to be a major reason why they decided to endure with IPV.

Participants shared that when they weighed up the possibility of reporting their husbands, they feared their imprisonment as this would result in fatherless children. In this context, keeping silent was thought to ensure a ‘good’ childhood for their children and protect them from challenges that would have followed the decision to leave the abuser. Becoming single mothers and going without the support of their in-law-family translated into many challenges for our participants, including poverty and discrimination.

When the women reflected on this, they stressed their and their children’s economic dependence on the abuser. In this context, keeping silent was described as a strategy towards maximizing the survival of the woman and of her children. As a result, some participants decided to divorce only after their children were economically independent.

#### *Fear of being ashamed and blamed* (Table 3)

Another reason why women kept silent was their fear of stigma and the shame associated with IPV. If reported, the abuse these women experienced in their marital relationship would be made public and they would be blamed for their husbands’ incarcerations and its consequences. Participants discussed the social stigma that leaving their abuser would bring because of the strong and challenging social norms and stereotypes regarding unmarried or divorced women in Vietnam. This convinced them that the best strategy to protect themselves and their children was keeping silent, as this would protect them from gossip and discrimination. It is relevant to note that in their accounts about fearing shame and stigma, children’s wellbeing and protection was a priority for these women. Participants were concerned that their children would be ashamed if their friends discovered the IPV. Having a father with a criminal record could negatively affect children’s future because it could be difficult to find a job or even a wife. The women felt that as mothers, they needed to ensure a stable family environment for their children.

Participants shared that their relatives were aware of how the social stigma of leaving their abuser would affect them. Family members asked the women to remain with the abuser, and participants expressed that their families would talk about women that endured the violence as an example to follow. Women were reminded of their role as daughters-in-law, one which entails keeping the family’s secret following older women’s experiences or advice as well as protecting the honor and reputation of the families they married into. For some participants, the advice to follow the example of women that endured IPV, like their mothers, contributed to the normalization of the violence. The women in our study stated that their communities interpret IPV as a women’s failure to achieve the ideal of family harmony.

### Leaving the abuser

Participants’ accounts of ‘leaving the abuser’ included their discussions regarding the steps that they took during the process as well as their decision to leave after realizing that keeping silent would not ensure their survival or that of their children. All interviewees also shared their wishes of finding a job and having happy lives without their husbands.

#### *Seeking help from family and friends* (Table 4)

Among the choices participants considered in order to deal with their situation, being able to approach their local help system like relatives, friends and neighbors stood out. However, this turned out to be a failed step in maximizing informants’ security because the abusers would ask relatives and friends to mediate between them and their wives or the abusers would threaten them. Because of the risk of violence that the abuser would present to anyone helping his wife, women were only offered a temporary place to stay, one that could only be used for a short time. This was not an option for the women who had moved to their husband’s city, as they lacked social networks and local social support nearby.

#### *Seeking help from the PHS* (Table 5)

Participants would consider seeking help from PHS when they experienced the failure of their local support system to protect them and their children from the abuser. Yet running away and breaking the silence was not easy. On the contrary, this involved some risks. For instance, if the husband found out that the woman wanted to run away, the woman could face even more violence and punishment.

The women explained that they left their abuser when they had given up any hope that the violence would ever end. They reached this decision after a long period of abuse and attempts to live with their violent husbands. Participants reported experiencing increasing levels of IPV over time, which convinced them that their relatives’ advice about being silent could not ensure their safety nor foster changes in their husbands’ behavior. For the participants coming from rural areas, seeking help from PHS was more difficult because they would need to move to the city where a shelter is located. This increased their perceived uncertainty because they would need to find a job and a school for their children, as well as burden them with having to get to know an unfamiliar place. Moreover, the lack of information about the supportive services and the rights they were entitled to as victims of IPV would add more anxieties and uncertainty while taking the step of seeking help.

A common reason why women decided to seek help from the shelter was their children’s wellbeing. Participants narrated that as the violence became more unbearable, they realized that it could have a negative impact on their children who were victims of the abuse either indirectly or directly. This fear came up strongly when women witnessed that IPV brought changes in their children’s behavior, personality, health, and school performance. Particularly, they realized that their children were acting angry, rude, and violent towards others. Finally, escaping from violence and seeking help from the shelter could result in the decision to divorce the abusive husband. This was the most decisive step to put an end to IPV. However, making this decision was difficult for participants, especially due to their limited knowledge of marriage and domestic violence laws, as well as child custody and property division. In this regard, the support from the PHS staff was of great help in informing the women about divorce procedures. The staff from the PHS would also teach women some tactics to maximize their protection when they would meet their husbands during the divorce procedures.

#### *Seeking and taking others’ advice* (Table 6)

The strategy of leaving the abuser was also motivated by the advice women sought and received from others who were critical to cultural stereotypes and gender roles according to which women should remain silent. Our informants valued this advice from women who had experienced or witnessed IPV in the past. This could come from in-laws and mothers who, based on their own experience of IPV, would encourage their daughters to put an end to the abusive relationship. To a lesser extent, advice on seeking help from the PHS could also come from strangers or acquaintances.

## Discussion

Participants used two strategies to maximize their survival: to bargain patriarchy by keeping silent and staying with the abuser; or to put an end to IPV by seeking help and leaving their husbands. Women described these as two different survival strategies to deal with IPV. In some cases, these were used sequentially and made for a two-step process that helped women to put an end to the abusive relationship. For others, these were not concurrent but separate strategies. This was the case for women who left the abusive relationship permanently after a process of repeated incidences of leaving and returning to their abuser. It is important to highlight that women’s accounts on bargaining with patriarchy came accompanied by their discussions of their personal motivations for using this strategy. However, when informants reflected on their decision to leave the abuser, they did not focus on what was the motivation behind this decision, but on the steps involved in such strategy. We argue that this has to do with the help-seeking context in which the interactions between researcher and informants took place. All participants had sought help from the PHS and had established user-professional relationships with the staff from whom they received professional intervention. Thus, we assume that informants were familiar with therapeutic interactions in which they were asked to make sense of IPV, reflect on the reasons why they did not seek help earlier, and stress their agency in their decisions to leave the abuser.

Framed by the theoretical concept of patriarchal bargain [14], we discuss that the women strategized within a complex set of structural constraints- poverty, traditional gender roles, social stigma, cultures of honor- aiming to optimize their survival within the Vietnamese system of male dominance. Consistent with previous studies [24–26], the results show that motherhood plays a decisive role in the decisions women make regarding whether to bargain with patriarchy or leave the abuser. Their children’s wellbeing, understood as a state of physical and emotional health but also social inclusion and belonging, was paramount for the participants and was at the core of their experiences. Keeping silent came up as a strategy that would bring more benefits than that of challenging the patriarchal system. In particular, children’s basic needs for food and shelter, an upbringing with both a mother and a father figure, and their protection from social stigma, were elements that the women considered. Economic dependence from the abuser arose as an important barrier that hindered mothers from challenging the patriarchy. At the same time, their children’s wellbeing could also motivate women to seek help and leave the abuser, and was mentioned as a key concern.

The authoritative role of senior women, which is a central feature of the societies within the patriarchal belt like Vietnam [14], was very relevant regarding our informants’ strategizing for their security. Our findings show how mothers and mothers-in-law may decide to take a conformist stance and remind our informants about their duty to protect the family’s honor and reputation by enduring the abuse. In doing this, senior women maintained an order of female subordination that secured their survival and their higher better status within the patriarchal system. We argue that these privileges are the reason behind the advice these senior women gave our participants, particularly when it was related to tolerating the abuse to a greater extent than their mothers, who more often adviced against staying. This mirrors the complex context in which our informants strategized for their survival, one where the strong ideal of family harmony fell on their shoulders [18,19].

In the Vietnamese context of male dominance, our participants were active agents who may have decided not to challenge the patriarchal system when this would optimize their life choices in the face of oppression. Factors like lack of knowledge about their rights and available services, and lack of social support, were decisive in their decisions to bargain with patriarchy. These were especially present in the accounts of women coming from rural areas to the city, as they required to move from their current geographical location when seeking help from the PHS.

The women in our study may have been motivated to leave their abuser and challenge the patriarchal system by their repeated experiences with abuse, which convinced participants that the situation of IPV would not stop in the future. In addition, access to formal or informal social support may have contributed to their decisions. This was a challenging strategy that required the help and support from others, something that was difficult to get due to the social stigma associated with divorce and the normalization of violence in intimate relationships [27] Drawing on the IPV stigmatization model [28], we claim that participants faced cultural and anticipated stigma. The former refers to those societal beliefs framed by the Confucian philosophical tradition that delegitimize women experiencing abuse. The latter involves those concerns the women shared in the interviews about the negative consequences of disclosing IPV, like rejection and social stigma. In this context, social support plays a key role in women’s decisions regarding IPV reinforcing or diminishing social stigma [10].

## Trustworthiness

Credibility was ensured by carefully describing the method, systematically analysing the data; by the multidisciplinary nature of the author team, which included anthropologist, public health specialists, and gender scholars; and by presenting quotes to demonstrate that the analysis is grounded in the data [23]. Transferability was enhanced by giving a detailed description of the study context, participants, and data collection and analysis. By doing so, we aim to help readers to assess whether these findings are applicable in similar contexts [29]. As for dependability, we followed an emergent design meaning that the insights were the result of interactions with the PHS staff and residents, and that these were adapted to the research. Moreover, ANT is a fluent Vietnamese speaker and conducted the interviews. This allows for consistency throughout the data collection process. Her ‘insider’ position encouraged a level of trust and openness in participants, which was relevant for their recruitment and the collection of rich data [30]. To avoid potential biases that this ‘insider’ position might have implied, reflexivity was included at all stages of the study and discussions with the rest of the authors, who were less familiar with the Vietnamese context, were crucial.

## Strengths and limitations

Some limitations of our study should be considered. First, selection bias may have taken place since the study only included women who were residents at the PHS. Second, IPV is a sensitive topic, which may pose difficulties for data collection. To overcome this, ANT received training from PHS and developed a good relationship with its staff and the interviewees. Moreover, drawing on her insider knowledge of the Vietnamese culture, ANT decided not to ask participants about their educational background to promote a comfortable atmosphere and avoid a reinforcement of the social distance between her and interviewees. Despite not having collected this demographical information directly, out participants’ occupation can give a picture of their social class and educational backgrounds (Table 1).

The strength of this study lies in that it provides rich data on the strategies that Vietnamese women use to cope with their abusive husbands. This knowledge will facilitate an improved understanding of IPV that is crucial for improved public health policy in the country. Because family harmony and social support played a key role in participants decision-making regarding the abusive relationship, we argue that addressing IPV in Vietnam requires holistic interventions that address structural barriers, such as poverty, and social stigma, and acknowledge women’s agency.

## Conclusion

Based on interviews with women survivors of IPV, this study contributes to a better understanding of this problem in Vietnam. It shows that both women’s decisions to endure the abuse or to leave the abuser are part of an active decision-making process in which they consider complex structural barriers. Among them, poverty, social stigma, discrimination, and cultural beliefs around the ideal of family harmony stand out. Motherhood is also a key factor that shapes women’s strategies. To better address IPV, interventions should be sensitive to the structural gender inequalities within family and societal contexts.

## Supplementary Material

Supplemental Material

## Paper context

Figures for IPV in Vietnam are high, and most survivors do not seek help from public services. This study shows the two main strategies IPV-survivors take to maximize theirs and their children’s security: keeping silent and/or leaving the abuser. In this decision-making process, the women weighed complex structural factors that are a barrier for them to escape the abuse. To better address IPV, interventions should be sensitive to structural gender inequalities within family and society.
