# Supplementary material for: Keeping silent or running away. The voices of Vietnamese women survivors of Intimate Partner Violence
Source: Glob Health Action. 2020 Dec 30;14(1):1863128. doi: 10.1080/16549716.2020.1863128 (PMC7781891; doi:10.1080/16549716.2020.1863128)
Supplement: Supplemental Material [file ZGHA_A_1863128_SM3885.pdf]

Online supplementary material: Example of qualitative content analysis process

Meaning unit	Condensed meaning unit	Code	Category	Subtheme	Theme
I was afraid he'd be in jail again. It's true that wife-beating breaks the law. Therefore, I was afraid. He had already stayed in prison one time, so it was easy for him to be put back again, and that would bring shame to my family again. […] Also my parents were ashamed of what people said about my marriage. And I felt shamed with everyone. I also did not share anything with my friends. (Resident 8).	My family and I were ashamed because people gossiped about my marital situation. If he goes to jail, we would feel shame too.	Social stigma associated with divorce that brings shame to the women and their families.	Cultural beliefs around family and marriage as a barrier to leave the abuser	Fear of being ashamed and blamed	Keeping silent
I think it [keeping silent] is because of worrying for their children and thinking of their families. We [women suffering from IPV] also think of our honor. We have a reputation as married women. I have to say that if we divorce, we would feel shame in front of other people. (Resident 7)	If we divorce, we would feel shame and bring shame to our families	Divorce brings stigmatization of the women and their children			
I had lived with violence for a long time in the past. I had seen my father beat my mother. So, it affected me […] […] I convinced myself that my husband could beat me.<br>[…]<br>When I told my father (about the abuse), he said, "He is a husband, so he has the right to beat and scold his wife". (Resident 1)	I had normalized abuse.<br>A husband has the right to beat his wife.	Normalization of IPV and stigmatization of women who do not endure it.			
